# Ribosomal dysregulation: A conserved pathophysiological mechanism in human depression and mouse chronic stress

**DOI:** 10.1093/pnasnexus/pgad299

**Published:** 2023-10-10

**Authors:** Xiaolu Zhang, Mahmoud Ali Eladawi, William George Ryan, Xiaoming Fan, Stephen Prevoznik, Trupti Devale, Barkha Ramnani, Krishnamurthy Malathi, Etienne Sibille, Robert Mccullumsmith, Toshifumi Tomoda, Rammohan Shukla

**Affiliations:** Department of Microbiology and Immunology, Louisiana State University Health Sciences Centre, Shreveport, LA 71105, USA; Department of Neurosciences, College of Medicine and Life Sciences, University of Toledo, Toledo, OH 43614, USA; Department of Neurosciences, College of Medicine and Life Sciences, University of Toledo, Toledo, OH 43614, USA; Department of Medicine, College of Medicine and Life Sciences, University of Toledo, Toledo, OH 43614, USA; Department of Neurosciences, College of Medicine and Life Sciences, University of Toledo, Toledo, OH 43614, USA; Department of Biological Sciences, College of Natural Sciences and Mathematics, University of Toledo, Toledo, OH 43614, USA; Department of Biological Sciences, College of Natural Sciences and Mathematics, University of Toledo, Toledo, OH 43614, USA; Department of Biological Sciences, College of Natural Sciences and Mathematics, University of Toledo, Toledo, OH 43614, USA; Centre for Addiction and Mental Health, Campbell Family Mental Health Research Institute, Toronto, ON M5T 1R8, Canada; Department of Psychiatry, University of Toronto, Toronto, ON M5T 1R8, Canada; Department of Neurosciences, College of Medicine and Life Sciences, University of Toledo, Toledo, OH 43614, USA; Neurosciences Institute, ProMedica, Toledo, OH 43614, USA; Centre for Addiction and Mental Health, Campbell Family Mental Health Research Institute, Toronto, ON M5T 1R8, Canada; Department of Zoology and Physiology, University of Wyoming, Laramie, WY 82071, USA

**Keywords:** orthologs, major depressive disorder, chronic variable stress, ribosomes, species and sex differences

## Abstract

The underlying biological mechanisms that contribute to the heterogeneity of major depressive disorder (MDD) presentation remain poorly understood, highlighting the need for a conceptual framework that can explain this variability and bridge the gap between animal models and clinical endpoints. Here, we hypothesize that comparative analysis of molecular data from different experimental systems of chronic stress, and MDD has the potential to provide insight into these mechanisms and address this gap. Thus, we compared transcriptomic profiles of brain tissue from postmortem MDD subjects and from mice exposed to chronic variable stress (CVS) to identify orthologous genes. Ribosomal protein genes (RPGs) were down-regulated, and associated ribosomal protein (RP) pseudogenes were up-regulated in both conditions. A seeded gene co-expression analysis using altered RPGs common between the MDD and CVS groups revealed that down-regulated RPGs homeostatically regulated the synaptic changes in both groups through a RP-pseudogene-driven mechanism. In vitro analysis demonstrated that the RPG dysregulation was a glucocorticoid-driven endocrine response to stress. In silico analysis further demonstrated that the dysregulation was reversed during remission from MDD and selectively responded to ketamine but not to imipramine. This study provides the first evidence that ribosomal dysregulation during stress is a conserved phenotype in human MDD and chronic stress-exposed mouse. Our results establish a foundation for the hypothesis that stress-induced alterations in RPGs and, consequently, ribosomes contribute to the synaptic dysregulation underlying MDD and chronic stress-related mood disorders. We discuss the role of ribosomal heterogeneity in the variable presentations of depression and other mood disorders.

**Significance Statement**

The presented study highlights the pressing need for a connection between animal models of depression and clinical endpoints. The lack of concordance between these two areas has hindered our understanding of depression's biological underpinnings. This study supports the hypothesis that orthologous genes from experimental systems of chronic stress and depression can bridge the gap between models of depression and the human condition, thus providing a major advance in the field. The study indicates that dysregulation of ribosomal protein genes is a common feature in both human depression and mice exposed to chronic stress. This dysregulation is a response to endocrine disturbances and can be linked to dysregulation of homeostatic synaptic scaling.

## Introduction

Major depressive disorder (MDD) is a debilitating mental illness that affects more than 300 million people worldwide and is ∼2 times more prevalent in females. Therapies and medication are available, but a 13% increased prevalence of MDD in the past decade suggests limited success of these treatments. This shortcoming between therapies and remission can be linked to the heterogeneous nature of depression presentation and our inadequate understanding of the disease biology. Human postmortem brain research has provided important insight into disease biology but is often associated with uncontrollable biological and environmental variables and undocumented medical histories. Thus, animal models of depression are a crucial tool for examining several molecular and cellular changes associated with depression in a controlled environment (1). For example, in rodents, chronic variable stress (CVS), a paradigm involving systematic and repeated exposures to variable, unpredictable, and uncontrolled stressors over days or weeks, recapitulates several features of MDD (2). However, in spite of the advances this (and other [1]) models have facilitated, a translational gap remains, largely due to the poor correlation between the aspects of depression explained by animal models and clinical endpoints. Many traits of human depression are either absent, difficult to assess, or radically different in animal models. Prior transcriptomic-based studies have attempted to identify similarities between rodent CVS and human MDD using integrated network-based analysis to identify a common hub gene for downstream analysis (3). However, such strategies often fail to reproduce reliable associations with disease (4). We hypothesize that identifying ortholog gene families, rather than hub genes, that are similarly affected between human MDD, and mouse CVS will provide seeds for gene network analyses, generating ontologies that uncover the structural and biochemical foundations of disease pathophysiology. Importantly, this strategy can be replicated using other data sets to highlight similarities and differences between species and sexes to further our understanding of the biological basis of disease heterogeneity and sexual dimorphism.

We initiated this study by identifying orthologous gene families altered in both the human MDD and mouse CVS conditions in existing (3) total RNAseq data from the prefrontal cortex (PFC; dorsolateral PFC [DLPFC] in human). Alterations in ribosomal protein genes (RPGs)—an evolutionary conserved family of genes—showed significant overlap between the two species. Our findings provide a foundation for a hypothesis proposing that stress-induced alterations in RPGs, which occur in species ranging from yeast to rodents and humans, result in ribosome dysregulation that may underlie pathophysiological changes observed in chronic stress and stress-related mood disorders. Importantly, the diverse infrastructure and mobility of ribosomes across neurites provide an opportunity for ribosome dysregulation to explain the immense variability in stress-response phenotypes and human depression subtypes.

## Results

### RPGs are dysregulated in the PFC of CVS-exposed mice and humans with MDD

**Fig. 1.**
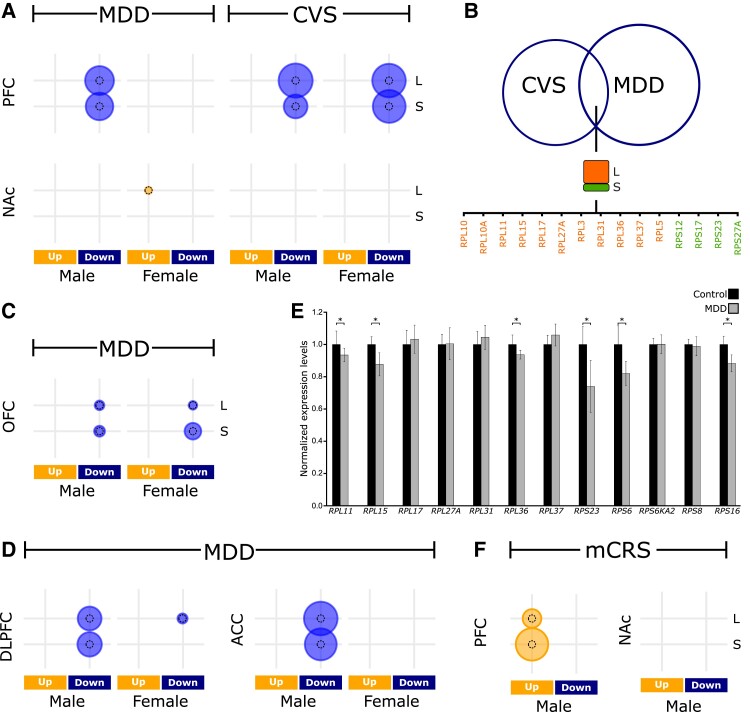
RPGs in the PFC are dysregulated during MDD and CVS. A) Enrichment of large (L) and small (S) subunit RPGs in the down-regulated genes in the PFC and NAc of humans with MDD and CVS-exposed mice. B) Overlap of down-regulated RPGs in MDD and CVS. C) Enrichment of RPGs in the OFC from the same cohort. D) qPCR results showing 6/12 common RPGs are down-regulated in the ACC of an independent cohort of patients with MDD. *n* = 6/group; *<0.05. E and F) RPGs were dysregulated during MDD (E) and mCRS (F) in prefronatal cortex data avilable from independent studies. The size of the circle is proportionl to −log_10_(*q*-value). The reference dotted circle is proportional to −log_10_(*q*-value = 0.05).

To identify gene family orthologs that are conserved in the DLPFC of humans with MDD and the PFC of mice exposed to 4 weeks of CVS, we performed differential expression analysis (Table S1) on reposited transcriptome data sets from previous work (3) and examined the enrichment of ∼1,200 Human Genome Nomenclature Committee (5) gene families in the sets of up- and down-regulated differentially expressed genes. We discovered significant enrichment of gene families associated with large and small RPGs in the down-regulated genes of male and female mice and male humans (Fig. 1A and Table S2). After harmonizing the gene symbols between the 2 species, we examined the overlap in the significantly down-regulated genes and identified 15 RPGs that were differentially expressed in the DLPFC of humans with MDD and the PFC of mice exposed to CVS (Fig. 1B, common RPGs henceforth).

Prior studies have shown that RPG expression varies by tissue type (6). Thus, we investigated the enrichment of large and small RPG families in the transcriptomic data for the nucleus accumbens (NAc; Table S1) of both species from the same cohort and analyzed additional data available from the frontal cortex region comprising the orbitofrontal cortex (OFC; Table S1) in the same human cohort. The NAc of neither species showed enrichment of RPG families (Fig. 1A, bottom, and Table S2). However, we did observe a significant enrichment of the RPG family in the down-regulated genes of the OFC for both males and females, albeit a less significant enrichment than the one observed in the DLPFC (Fig. 1C and Table S2). Analysis of independent reposited data sets (7) from the DLPFC and anterior cingulate cortex (ACC) of humans with MDD confirmed a significant enrichment of RPGs in down-regulated genes in both regions (Fig. 1D, Tables S1 and S2). qPCR analysis of human-specific differentially expressed RPGs in the ACC of male MDD and control subjects from an independent cohort also revealed significant down-regulation of several RPGs in the ACC (Fig. 1E).

To further validate our results in mice, we examined the differential expression profile of an independent data set from the PFC and NAc of mice who had undergone 3 weeks of multimodal chronic restrain stress (8) (mCRS; Fig. 1F), a stress paradigm with a different stress regime and allostatic load than the CVS paradigm. Consistent with the CVS results, enrichment analysis showed that RPGs were significantly dysregulated in the PFC but not in the NAc in mCRS-exposed mice (Fig. 1F and Table S2). However, unlike the CVS-exposed mice, the mCRS-exposed mice had up-regulated RPGs.

Overall, evidence from multiple independent studies demonstrates that gene families associated with ribosome structure are variable (i.e. either up or down) but significantly and consistently dysregulated during stress in mice and MDD in humans. As ribosomal proteins (RPs) constitute the ribosomes, these results point toward altered translation and translational machinery during stress.

### Seeded gene co-expression analysis reveals species- and sex-specific ribosome regulation

**Fig. 2.**
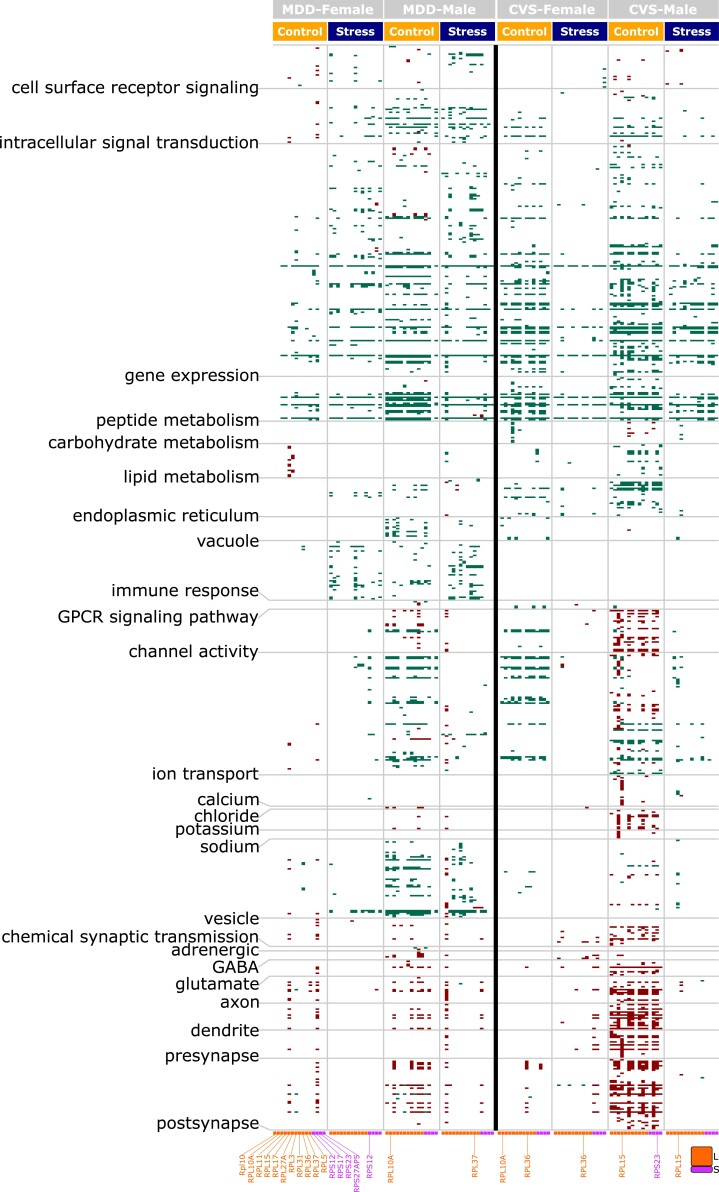
The MDD- and CVS-sentive changes in RPGs inversely regulate pathways associated with synaptic functions. A heatmap of common RPG-regulated pathways (q < 0.05) in both sexes during MDD and CVS phenotypes under control and stress conditions. Green and maroon: pathways positively and negatively regulated by RPGs, respectively. Notice the negative correlation of RPGs with intercellular communication-related themes and the variable number of RPGs associated with the control and stress state.

Next, we reasoned that, in a mechanism analogous to hub genes in gene co-expression network analysis, we could treat the common RPGs as seeds to identify correlated genes (Spearman correlation, *P* < 0.05; Table S3), thereby identifying the biological processes coordinated by the RPGs. Furthermore, we hypothesized that stratifying this seeded gene co-expression analysis for all conditions (i.e. stress/disease and control state in both sexes of both species) would reveal important sex- and species-specific differences in RPG-dependent regulation. The Gene Ontology (GO) terms associated with the RPG-correlated genes (*q* < 0.05) across all conditions were clustered into different themes (Fig. 2, left labels; Table S3), established in our previous meta-analysis of different psychiatric disorders (9, 10). Several intriguing associations stood out:

First, RPG-based regulation showed a striking contrast in the themes associated with positive and negative (shown as green and maroon, respectively) correlations. Nearly all intracellular events involving signal response coupling (*cell surface receptor signaling*, *intracellular signal transduction*, and *gene expression*), metabolic processes (*metabolism of peptides*, *carbohydrates*, and *lipids*), cellular organelles (*endoplasmic reticulum* and *vacuole*), and the immune response were positively correlated with RPGs. On the other hand, intercellular signaling events involving neuromodulatory infrastructure (*calcium*, *chloride*, *potassium*, *sodium*, *adrenergic*, g*amma-aminobutyric acid [GABA]*, and *glutamate*), neuromodulation activity (*G protein–coupled receptor signaling*, *channel activity*, *ion transport*, and *chemical synaptic transmission*), and synaptic infrastructure (*vesicles*, *axon*, *dendrite*, *presynapse*, and *postsynapse*) were negatively correlated with RPGs. As a negative correlation reflects a system's homeostatic balance (11), with inhibition of some genes and pathways being associated with the stimulation of others, the negative correlation of RPGs with synaptic alterations suggests a role for these common RPGs in homeostatic modulation of synaptic input and output.

Second, themes and pathways identified in the control state but absent in the MDD or CVS states suggest RPG coordination that was lost with stress or disease, and themes and pathways absent in the control state but present in the MDD or CVS states suggest new stress-altered coordination. Accordingly, females with MDD demonstrated increased intracellular events and decreased intercellular events related to RPG coordination compared with males, who exhibited only a marginal decrease in coordination across all themes in the MDD state. In CVS-exposed mice, however, this pattern differed, and both female and male mice exhibited decreased coordination across all themes in the stressed state. Notably, there was greater RPG coordination of the immune response in both sexes in the MDD population than in the controls, but no significant coordination of the immune response with RPGs was observed in CVS-exposed mice.

Lastly, among the 15 common RPGs (Fig. 1B) belonging to either the large or small subunit used for the analysis, a variable number and type (large or small) of RPGs were associated with the control and stress/disease states. Interestingly, the number of RPGs contributing to negatively correlated themes involving neuromodulatory infrastructure (*calcium*, *potassium*, *sodium*, *GABA*, and *glutamate*), fast neuromodulation activity (*channel activity*, *ion-transport*, and *chemical synaptic transmission*) and synaptic infrastructure (*vesicles*, *axon*, *dendrite*, *presynapse*, and *postsynapse*) decreased in the stress/disease condition in sex- and species-specific manners. For example, there was a decreased coordination of these themes in CVS-exposed male mice compared with control males.

Overall, the findings using the seeded gene network analysis imply that RPGs are responsible for the homeostatic feedback regulation of pathways associated with synaptic communication during both stress and MDD in both sexes. However, this regulation is differentially influenced by a diverse set of RPGs, indicating that ribosome composition may be changed in a sex- and species-specific manner.

### RPG dysregulation is likely governed by RP pseudogenes

**Fig. 3.**
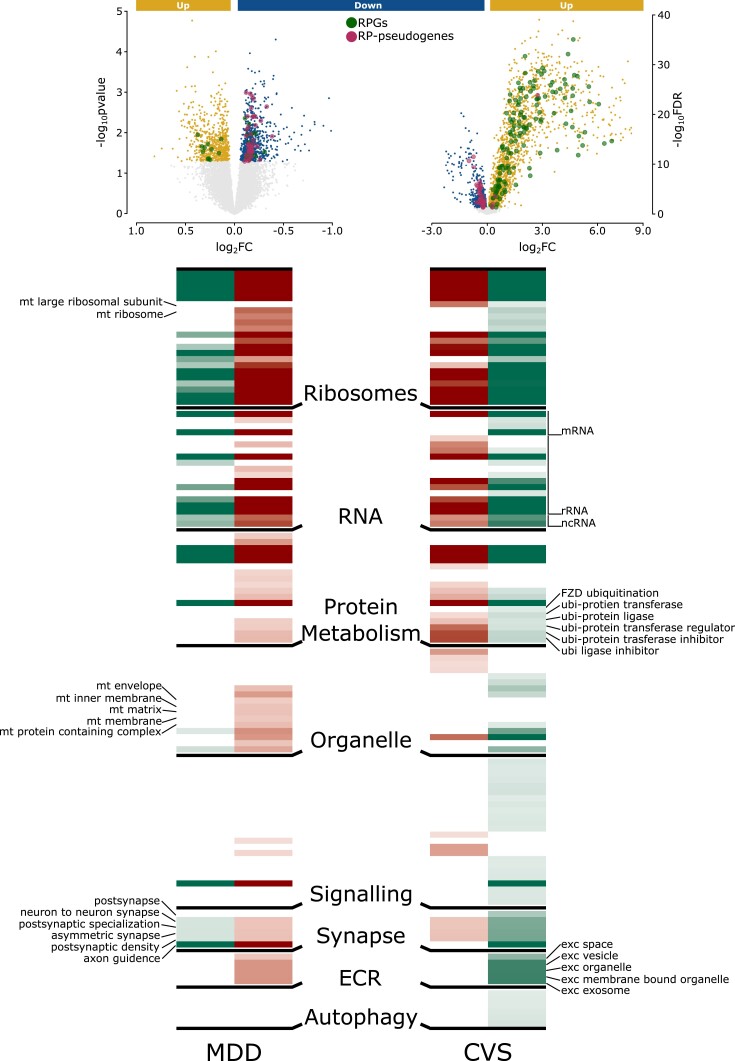
RPG down-regulation is regulated by pseudogenes: pathway profile associated with the down-regulated RPGs (maroon) and up-regulated RP pseudogenes (green) is shown. The pathways associated with both the RPG and RP pseudogenes are likely to be linked with an RP pseudogene during a stressed state. Notice that most of the housekeeping-associated pathways (*ribosomes*, *RNA*, and *protein metabolism*) and synapse-associated pathways associated with RPGs overlap with RP pseudogenes. The lighter to darker shade of both maroon and green shows increasing enrichment calculated as –log_10_(*q*-value).

RP pseudogenes are evolutionarily mutated DNA sequences that resemble RPGs and often regulate the expression and function of the parent RPGs. At the transcript level, regulation is either through RNA interference, where a pseudogene serves as a short interfering RNA to down-regulate parent RPG expression, or through the competitive endogenous RNA (ceRNA) pathway, where a pseudogene acts as a sponge for common micro RNAs (miRNAs) to attenuate down-regulation of parent RPG expression (12, 13). Notably, in mice and humans, RPG down-regulation was linked with RP pseudogene up-regulation (Fig. 3, top). This suggests that the identified down-regulated RPGs could potentially be regulated by pseudogenes. To determine whether RPG-associated functions are dependent or independent of RP pseudogenes, we mapped the ontologies (*q* < 0.05) associated with down-regulated RPGs (Fig. 3, maroon columns) and up-regulated RP pseudogenes (Fig. 3, green columns) to each other. There were phenotype-specific (CVS or MDD) differences in themes (Fig. 3, middle labels) associated with autophagy, extracellular region, signaling, organelle, and protein metabolism, and these themes showed minimal overlap with RPG- and RP-pseudogene-associated functions. However, canonical functions associated with ribosomes and RNA, as well as most synaptic pathways, revealed considerable overlap in RPG- and RP-pseudogene-associated functions during MDD and CVS, indicating that RP pseudogenes can selectively modify these RP-related functions in both species during stress and MDD.

Overall, RPG- and RP-pseudogene-specific functional analysis suggests that several of the dysregulated canonical and synaptic RPG functions may be regulated by pseudogenes.

### Remission from MDD is associated with RPG down-regulation reversal

Next, we reasoned that if RPG dysregulation is functionally associated with MDD, RPG down-regulation should be reversed during remission from MDD. To probe this hypothesis, we examined our existing data sets (14) that included data from the ACC of postmortem subjects who died during MDD episodes or in remission. Consistent with our core results, we observed down-regulated RPGs and up-regulated RP pseudogenes during MDD episodes. However, during remission, the down-regulated RPG and up-regulated RP pseudogene relationships observed during an MDD episode were reversed (Fig. S1). Furthermore, the canonical ribosome-related pathways (*q* < 0.05) were down-regulated during episodes and up-regulated during remission. To validate the accuracy of the episode and remission state, we examined their characteristic alterations. Episode state, consistent across several previous reports, was associated with down-regulated presynaptic and postsynaptic changes (15) and up-regulated glucocorticoid and immune response (16). Likewise, the episode and remission states, which are both defined as a depression trait, showed up-regulation of the innate immune response (14).

Overall, independent analysis of additional data sets confirmed the inverse association between RPG and RP pseudogenes, and analysis of the remission state indicated its functional association with MDD.

### RPGs are dysregulated in glucocorticoid-treated primary PFC neurons

**Fig. 4.**
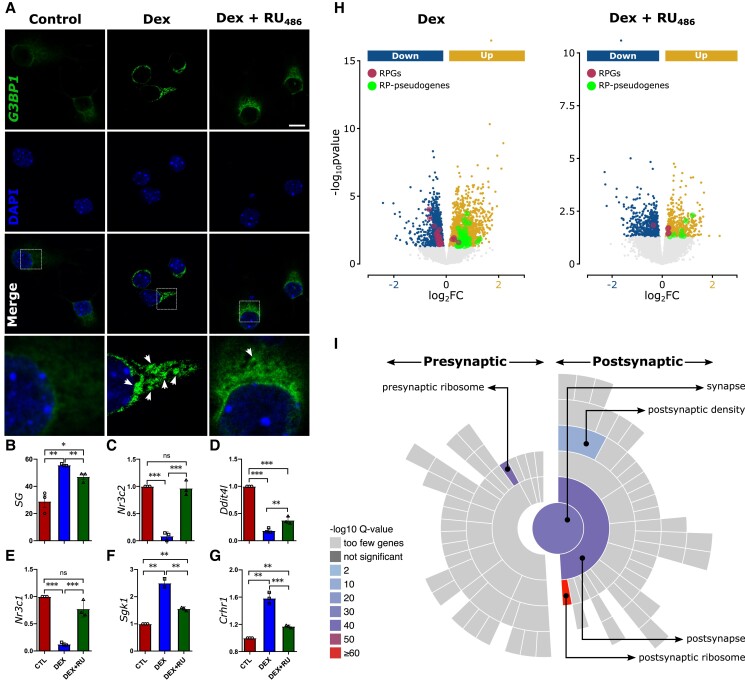
The inverse association between RPG and RPG pseudogenes is preserved in PFC primary neurons. A) DEX-treated PFC primary neurons displayed SG expression (white arrow), which is suppressed by RU_486_ treatment. B) Quantification of SG expression in control DEX and DEX + RU_486_ cells. Scale bar: 25 µm. C to G) The results of qPCR demonstrate that CVS markers are similarly dysregulated in glucocorticoid-stressed neuronal cells. *n* = 3/group. H) The inverse association between RPG and RPG pseudogenes observed during DEX is reversed and lost after RU_486_ treatment. *n* = 12/group. I) Similar to RP pseudogenes in the CVS and MDD conditions, the RP pseudogenes were significantly associated with synaptic pathways.

Both MDD (Fig. S1) and CVS (17) are associated with elevated levels of glucocorticoid activity, which are responsible for several of the negative consequences of chronic stress. Therefore, we reasoned that chronic treatment (72 h) (18, 19) of primary PFC neurons with a synthetic glucocorticoid, dexamethasone (DEX; 1 µM) (18), may replicate the inverse dysregulation of RPG and RP pseudogene expression observed during MDD and CVS. To verify stress induction in our in vitro model, we established that DEX-treated PFC cells showed prominent formation of stress granules (SGs), a putative marker of stress response (Fig. 4A and B) (20, 21). Additionally, qPCR analysis demonstrated that DEX-treated PFC cells expressed several known markers of chronic stress identified in experimental models (Fig. 4C–G) (22–27). We then compared the RNAseq-based expression profiles of control and DEX-treated primary neurons. Notably, consistent with the expression profiles of the patients with MDD and CVS-exposed mice (Fig. 3, top, and Fig. S1), we observed an inverse association between RPGs and RP pseudogenes in DEX-treated primary neurons that was attenuated when the cells were co-treated with DEX and RU_486_, a glucocorticoid receptor antagonist (Fig. 4H). Importantly, the up-regulated RP pseudogenes influenced pathways related to synaptic signaling themes (Fig. 4I).

Overall, the in vitro experiment confirmed the chronic stress-related endocrine origin of RPG dysregulation. Thus, across all three chronic stress paradigms (human MDD, mouse CVS, and glucocorticoid-stressed primary neurons), pseudogenes may play a vital role in regulating stress response pathways and synapse-associated RPG functionality.

### Ketamine, but not imipramine, alleviates RPG dysregulation

In both animal models and humans, stress exposure uncovers distinct groups that are susceptible to and resilient against perturbations caused by stress. Additionally, stressed individuals can be classified as responders or nonresponders to antidepressant treatment. To investigate how RPGs are dysregulated in these different phenotypes, we analyzed data from resilient and susceptible mice subjected to chronic social defeat stress (CSDS), a stress paradigm with a different allostatic load than CVS, as well as responders and nonresponders to imipramine and ketamine treatments, all within the same cohort (Fig. S2 and Table S4). Consistent with the CVS-based findings, we found a significant down-regulation of RPGs in resilient mice. However, RP pseudogenes were up-regulated in susceptible mice but not in resilient mice. In the groups treated with antidepressants, ketamine attenuated the expression of both RPGs and RP pseudogenes in both responder and nonresponder animals. Surprisingly, however, imipramine did not lead to any alterations in RPG or RP pseudogene expression in either the responder or the nonresponder groups.

Overall, our analysis revealed differences in RPG dysregulation among the resilient and susceptible phenotypes and in response to antidepressant treatment of differing modes of action. Given the established role of ketamine in triggering homeostatic synaptic upscaling (28, 29), the attenuation of RPG expression by ketamine supports the observed involvement of RPG dysregulation in the process of homeostatic regulation.

## Discussion

The RPG family, comprising more than 110 members, represents a highly diverse group of genes responsible for ribosome formation. Intriguingly, ribosomes play a vital role in the stress response across various phyla, with their stress-related functions extensively studied in simpler organisms like bacteria and yeast. Here, in accordance with their evolutionarily conserved involvement in stress, we demonstrated the involvement of RPGs in human MDD and mouse chronic stress, confirming our results through the analysis of multiple independent cohorts. Providing orthogonal validation for our findings, proteomics studies in the PFC of rats undergoing chronic stress (30) also revealed the dysregulation of several RPs (Fig. S3). Notably, amid the consistent dysregulation of RPGs across several stress paradigms and animal models, we observed phenotype-specific variations in the number and type (small or large) of RPGs that were differentially expressed. Nonetheless, in addition to the canonical ribosomal pathways, the observed dysregulation across all the phenotypes was consistently linked with neurite-related pathways, potentially providing a pathophysiological basis for the synaptic dysregulation underlying MDD and the chronic stress response. Typically, ribosomes, which are composed of RPs, are assumed to be homogeneous entities that translate the transcriptome uniformly. However, functional ribosome assembly is tightly controlled by the gene dose (i.e. mRNA levels) of RPGs; thus, the observed dysregulations of RPGs can result in a substoichiometric production of RPs, leading to the formation of heterogeneous ribosomes lacking certain RPs. Based on our findings, we propose a ribosome hypothesis for stress-related mood disorders: Stress-induced changes in RPs alter the number and composition of ribosomes, resulting in stress-induced ribosome heterogeneity impacting the synthesis of alternate proteins, which in neurites can shape neuronal information input and output, resulting in many forms of synaptic dysregulation that manifest as mood disorder symptoms. In our subsequent discussions, we will explore how the differential regulation of RPGs may be related to stress and stress-related disorders such as MDD.

To encode and learn complex patterns, each cortical neuron must access many possible combinations of axonal input (Fig. S4). These inputs can target various parts of the neuron, including the soma, distal dendrites, and proximal dendrites. Several psychiatric disorders are linked to location-specific dysregulation of inputs. For instance, dendritic input is reportedly altered during MDD (10), and somatodendritic input during schizophrenia (31). Our hypothesis posits that ribosomes, along with their diverse RPs, sculpt the adaptability of neurons to variable inputs through the following potential mechanisms (Fig. S4): First, location-nonspecific down-regulation of RPGs can lead to decreased ribosome biosynthesis, thus reducing global translation and protein synthesis. Ribosome biosynthesis is an energy-intensive process that requires several essential amino acids and the presence of adenosine triphosphate. Consequently, even simple species such as *Saccharomyces cerevisiae* have a homeostatic mechanism during stress that halts ribosome biosynthesis via down-regulation of RPGs to conserve energy (32). RPG down-regulation in human MDD and mouse CVS may preserve several essential amino acids, such as lysine and arginine, which are abundant in ribosomes (33, 34) and whose deficiency has been functionally linked to depression (35–37). In neurons, most RPGs are found in neurites; thus, the homeostatic down-regulation of RPGs will decrease synaptic protein synthesis. Interestingly, this appears to be consistent with the process of homeostatic synaptic scaling, a plasticity mechanism in which a neuron regulates its own excitability in relation to network activity, primarily by targeting the protein synthesis process. Indeed, males in the human MDD and mouse CVS groups exhibited an excessive loss in coordination of pathways associated with glutamatergic singling (Fig. 2). Further support for the role of RPG dysregulation in maintaining homeostatic synaptic scaling comes from the following observations: (i) the inverse association between somatic and neuritic pathways linked with seeded RPGs, which is consistent with the known inverse association between the expression of crucial regulators of homeostatic synaptic scaling in somatic and neuritic compartments of neurons (38), and (ii) the selective attenuation of RPG and RP pseudogene dysregulation during ketamine treatment (Fig. S2), which has an established role in triggering homeostatic synaptic upscaling (28, 29). The mechanisms of homeostatic synaptic scaling are largely unknown. Our results point toward RP pseudogenes as potential candidates driving the homeostatic down-regulation of RPGs (Fig. 3). Notably, unlike long-term potentiation or depression, where synaptic strengths are rapidly potentiated or depressed, synaptic scaling develops over several hours to days (39) and is dependent on protein synthesis. The average half-life of pseudogenes (10 to 17 h) (29) falls into the range of duration required for homeostatic scaling, further supporting the possible role of RP pseudogenes in mechanisms of homeostatic synaptic scaling.

Homeostatic synaptic scaling can operate at a local level by targeting a group of synapses on a certain dendritic branch or at a synapse-specific level (28). Following that, the second mechanism by which ribosomes can sculpt the adaptability of neurons to variable inputs is through a site-specific modification of ribosome composition. Ribosome heterogeneity allows for specialized ribosomes that provide preferential translation regulation (40). Specialized ribosomes can have distinct modifications with functional outcomes ranging from changes in translation initiation, speed, fidelity control, and mRNA translation selectivity (Fig. S4). Functional specializations of ribosomes may also result from activity-dependent phosphorylation (41) and other posttranslational modifications (42) on selected RPs, thereby further modifying ribosome composition. Future studies will explore stress-induced ribosome specialization and its functional consequences for neuronal input and output and synaptic dysregulation.

We observed different directionalities in RPG expression changes in various chronic stress paradigms, each with distinct stress regimes and allostatic loads. Unlike Hebbian plasticity, which operates in a feedforward manner to align changes in synaptic strength with applied stimuli in the same direction (43), homeostatic synaptic scaling functions through negative feedback to restore neuronal activity patterns to their initial set point by adjusting synaptic strengths in the opposite direction (44, 45). Therefore, the directionality of dysregulation likely depends on the specific neuronal activity patterns associated with different stress paradigms. This intriguing observation suggests that RPG dysregulation and its directionality could serve as markers of neuronal activity in response to different stress paradigms, offering a promising avenue for future research.

The two mechanisms by which ribosomes can sculpt the adaptability of neurons to variable inputs—location-nonspecific RPG down-regulation and site-specific modification of ribosome composition—have several implications for mood disorders and therapeutic strategies targeting them. First, depression can be viewed as part of a spectrum of mood disorders, with several types of depression lying along a continuum with nebulous boundaries between them. Dendritic input is dysregulated during depression, resulting in a remodeling of the microcircuitry associated with learning, memory, and attention (10). Together, location-nonspecific RPG down-regulation and site-specific modification of ribosome composition can result in several permutations supporting dysregulation of synaptic input along the dendritic arbor. These permutations can contribute to nuances resulting in the spectrum nature of depression and other mood disorders. Second, targeting RP expression regulation and its role in ribosomal heterogeneity offers a promising therapeutic strategy previously unexplored. Ribosomes are typically associated with protein synthesis, which in dendrites can involve transmitter receptors, synaptic scaffold proteins, and other regulatory proteins. Thus, our novel ribosome hypothesis can encompass other known hypotheses of depression involving GABA (46), glutamate (47), immune (48), and monoaminergic (49) systems. As such, exploring the therapeutic options associated with ribosomal dysregulation may have a broad transdiagnostic impact across depression subtypes.

## Materials and methods

### Data cohorts

We downloaded data sets in the fastq.gz format from Gene Expression Omnibus (GEO) with ID GSE102556 (3), which included both mouse and human data sets for CVS and MDD, respectively. Mouse data were available only from the PFC (female = 19, male = 19) and NAc (female = 20, male = 20). For humans, data were available from several brain regions, but we focused on analyzing the regions homologous to the available mouse regions. This included the DLPFC (female = 22, male = 26, mean age = 47), OFC (female = 22, male = 26, mean age = 47), and basal forebrain region involving the NAc (female = 22, male = 28, mean age = 47). To validate our findings, we used additional data sets from mice subjected to a multimodal chronic restraint stress (mCRS) paradigm (GSE148629) and humans with MDD (GSE80655). GSE148629 had both mouse PFC (*n* = 15, age = 15 weeks) and NAc (*n* = 8, age = 15 weeks) data from males. GSE806558 had MDD data from the human DLPFC (female = 6, male = 17, average age = 46 years) and ACC (female = 6, male = 16, average age = 45 years), which is also a PFC region. We validated the ACC-related findings using our previous study (14) and qPCR analysis described in the subsequent methods section. To understand RPG dysregulation in the PFC of mice with a resilient (*n* = 4) or susceptible (*n* = 3) phenotype and an imipramine and ketamine responder or nonresponder phenotype (*n* = 3 for each), we used a data set from male mice subjected to a CSDS paradigm (GSE81672) (50).

### Alignment protocol

We used HISAT2 version 2.1.0 to index mouse and human reference genomes and align downloaded RNAseq data. We then used subread version 1.5.0-p2 to quantify reads and map them to genomic features. Notably, RPGs have about 2000 RP pseudogenes that are significantly homologous to parent RPGs (51), which impedes the ability of the standard alignment method to uniquely identify reads mapped to RPGs and RP pseudogenes with high sequence homology. To circumvent this, we used a specialized alignment method as described before (52). Briefly, we mapped all sequence reads to a “composite genome” that includes the entire human (GRCh38) or mouse (GRCm38) genome sequence and spliced mRNA sequences of RPGs (52). We then mapped RNA-seq reads to the composite genome without allowing mismatches and discarding reads mapped to more than one locus, thus ensuring that the reads mapped to RP pseudogenes are not from repetitive regions or normal RP genes.

### Differential expression analysis

After removing the low-count genes (mean row sum ≤5 counts across all samples), we used DESeq2 in R to assess the differential expression of the remaining genes (Table S1) in comparisons between the control and human MDD groups and between the control and chronic stress-exposed mouse groups, divided by sex. For the GSE102556 and GSE80655 data sets, we used the SVA package in R to identify two surrogate variables that accounted for an unknown source of variation in all MDD-related contrasts (i.e. the comparison between MDD and control states in both sexes). We identified these variables by creating a full model matrix that included adjustment variables and variables representing the phenotype (i.e. control and MDD groups), as well as a null model that contained only adjustment variables. Depending on the data set, the adjustment variables included age, RNA integrity, postmortem interval, and pH, which were prioritized based on the total variability they explained as determined by the variance partitioning package in R (see Fig. S5). We used a *P*-value threshold of 0.05 for all MDD-related contrasts and a false discovery rate (FDR)-corrected *P*-value (*q*-value) threshold of 0.05 for all CVS-related contrasts.

### Enrichment of gene families in differentially expressed genes

To identify the stress-related ortholog genes shared by the two species (CVS: mouse and MDD: human), we performed hypergeometric overlap-based enrichment analysis implemented by the GeneOverlap package in R-3.6.0. The significance of the overlap between two gene lists can be tested using a hypergeometric distribution performed with a genomic background representing the universe of known genes (21,196 genes, the default used by the package). The GeneOverlap class formulates the problem as testing whether the two gene sets are independent and then uses Fisher's exact test to find the statistical significance. The significant overlap (*q* < 0.05) of the differentially expressed genes identified in the MDD and CVS contrasts was tested against gene family-specific gene sets (Table S2) common to both species available from the Human Genome Gene Nomenclature Committee (5). To compare the effect of gene families across the different MDD and CVS contrasts, the −log10(*q*-value) was used.

### Pathway enrichment analysis

To identify the biological pathways affected by the down-regulated RPGs and up-regulated RP pseudogenes, we searched for the enrichment of different GO terms associated with the identified RPGs and RP pseudogenes in the biological pathway, molecular function, and cellular component categories using the GO database. RP pseudogene symbols for both species resemble their respective parent RPG symbols, except for an additional suffix “-ps” for mouse and “AS” for human, which we removed to perform RP-pseudogene-specific enrichment analysis. Only FDR-corrected pathways (*q* < 0.05) were considered for subsequent analysis. To reduce and catalog the long list of GO terms into an interpretable format, we clustered the pathways based on biological themes as described in our previous works (10, 14, 53, 54). Briefly, the theme for a given pathway was selected based on either a text search, in which the name of the theme was used as a keyword for the text query, or on the parent–child association between the GO terms in our list of significant pathways (child pathways) and the handpicked pathways in the GO database (parent pathway) representing the theme using GOdb in R.

### Seeded gene network analysis

The down-regulated RPGs shared by the mouse and human data sets were considered “seed RPGs” and used to find Spearman correlation with the expression profiles of individual genes in the normalized CVS and MDD data sets (Table S3). Genes both negatively and positively correlated with a seed RPG were identified using a *P*-value threshold of <0.05 and used to perform pathway enrichment analysis as described above. Both the *r*- and *P*-values were computed using the cor.test() function in the stats R package.

### Primary culture and neuron treatment

Dissociated embryonic day 18 (E18) mouse cortical cells (C57EDCX, BrainBits) were obtained from BrainBits (Springfield, IL, USA) in 2 mL NBactive1 medium (2% B27 and 0.5 mM Glutamax). We transferred the cell medium (1 mL) into a 10-cm plate, triturating (5×) it through a sterile P1000 micropipette tip. We dissociated the cells in the remaining 1 mL medium via trituration (5×) in the vial. We combined the triturated media (2 mL) in a 15-mL conical tube, followed by centrifugation for 1 min at 1,100 rpm (220*g*). We aspirated the supernatant with a pipet (5 mL), resuspended the cell pellet in NBactive1 medium (NB1, BrainBits), and triturated and counted the cells. We diluted cells to 1.75 × 10^5^/mL following the manufacturer's protocol and plated 2 mL of cell suspension on a 6-well (3.5 × 10^5^) poly-D-lysine-coated plate (354413, Corning). Cells were incubated in a humidified environment at 37°C, 5% CO_2_ for 10 days with half of the growth medium replaced every 4 days. We prepared stock solutions of 1 mg/mL of DEX (D4902, Sigma-Aldrich) a glucocorticoid receptor agonist and 1 mg/mL mifepristone (RU-486, M8046, Sigma-Aldrich), a glucocorticoid receptor antagonist, following the manufacturer's protocol. On the eleventh day in vitro, the growth medium was aspirated and replaced with DEX medium (2 mL, 1 µM final concentration of DEX) (18, 55), DEX and RU-486 medium (2 mL, 1 µM final concentration of DEX and RU-486, respectively), or fresh growth medium, and incubated in the same environment for 72 h. The treatments were replenished at 48 h posttreatment.

### RNA extraction and sequencing

Using the RNeasy Mini Kit (Cat. 74104, Qiagen), we isolated total RNA from 12 experimental replicates of the DEX, DEX + RU-486, and control groups, for a total of 36 samples, according to the manufacturer's instructions. DNase I treatment (Cat.79254, Qiagen) was performed during the RNA isolation procedure to remove genomic DNA, following the manufacturer's recommendation. Total RNA was quantified using the NanoDrop One (Thermo Scientific, USA) and the Agilent Tape Station. We prepared a total RNA library for each sample, performed paired-end next-generation RNA sequencing with a depth of 60 million reads per sample on the NovaSeq platform (Illumina) at the University of Michigan Advanced Genomics Core, and processed the fast.qz files using the pipeline described above.

### Immunofluorescence analysis

To evaluate SG formation, we quantified the expression of the SG protein, G3BP1. We cultured mouse cortical neurons on poly-D-lysine-coated glass coverslips (Neuvitro, WA, USA) for 10 days, replacing half of the growth medium every 4 days. We treated the neurons with DEX or DEX + RU-486 for 72 h, fixed them with 4% paraformaldehyde (Boston Bioproducts, MA, USA) for 15 min, and permeabilized them with 0.2% Triton X-100 in phosphate-buffered saline (PBS) for 15 min. Cells were then blocked with 3% bovine serum albumin (BSA) and 0.02% Tween 20 for 1 h at room temperature and incubated overnight at 4°C with anti-G3BP1 antibodies (1:250; SC-81940, Santa Cruz Biotechnology, CA, USA), followed by a 1-h incubation with an Alexa488-conjugated anti-immunoglobulin secondary antibody (Molecular Probes) at room temperature. Cell nuclei were stained with Fluoro-Gel II with DAPI (EM Sciences, PA). We performed fluorescence and confocal microscopy assessments using a Leica CS SP5 multiphoton laser scanning confocal microscope (Leica Microsystems) and all subsequent image analysis and processing using the Leica application suite AF software (Leica Microsystems). Cells containing SG (*n* > 5) > 0.6 µm in diameter were considered for analysis. The percentage of SG-containing cells was calculated in at least 5 random fields from a minimum of 30 cells per treatment. Subsequent image analysis and processing were performed using Image J software (NIH, MD, USA). We assessed the statistical difference in SG formation between control, DEX, and DEX + RU-486 using one-way ANOVA, with a *P*-value of <0.05 considered statistically significant.

### Quantitative PCR

We performed a qPCR assay on primary cell culture samples, which included control cells, cells treated with DEX, cells treated with DEX + RU-486, and human postmortem ACC samples, which included control and MDD subjects. Gene-specific primers for both mouse and human genes, along with the endogenous control gene *Actb*, were ordered from Integrated DNA Technologies, Inc. (IDT, IA, USA), and the primer sequences for all analyzed genes are provided in Table S5. For the primary cell culture assay, we selected five genes as markers for CVS-exposed mice based on a literature review. However, due to the significant sequence homology between RPGs and their RP pseudogenes, which posed a challenge in designing RPG-specific primers, we used 12 human-specific RPGs, some of which were part of the common RPGs. For primary cell-culture samples, total RNA was isolated using Trizol reagent (Invitrogen), following the manufacturer’s instructions for cells cultured on poly-D-lysine-coated glass coverslips. We performed reverse transcription and cDNA synthesis using oligo-dT primers and RevertAid Reverse Transcriptase (Thermo Fisher Scientific). Gene expression for the five marker genes was quantified by qPCR using SYBR Green PCR Master Mix (Bio-Rad Laboratories Inc., Hercules, CA, USA), in three independent samples per treatment. For human samples, cDNA from the human postmortem ACC of control and MDD subjects was obtained from the remains used for our previously published work (14), and gene expression for 12 RPGs was quantified using SYBR Green PCR Master Mix in 6 samples each for the control and MDD conditions. The statistical difference between control, DEX, and DEX + RU-486 treated primary cells was assessed using a one-way ANOVA, and the difference between human control and MDD was assessed using a one-tailed t test. A *P*-value of <0.05 was considered statistically significant.

## Supplementary Material

pgad299_Supplementary_Data

## Data Availability

The generated data sets of this study were made publicly available at the GEO repository (GEO accession number: GSE229905) and can be accessed via the following link: https://www.ncbi.nlm.nih.gov/geo/query/acc.cgi?acc=GSE229905. Codes for all bioinformatic-based analyses are provided on the associated GitHub page: https://github.com/MEladawi/Ribosome_paper.

## Preprint

This manuscript was posted on a preprint: https://doi.org/10.1101/2023.05.04.539419.
