# Supplementary material for: Ribosomal dysregulation: A conserved pathophysiological mechanism in human depression and mouse chronic stress
Source: PNAS Nexus. 2023 Oct 10;2(10):pgad299. doi: 10.1093/pnasnexus/pgad299 (PMC10563789; doi:10.1093/pnasnexus/pgad299)
Supplement: pgad299_Supplementary_Data [file pgad299_supplementary_data.zip › PNASNEXUS-PNASNEXUS-2023-00398RR-s07.docx]

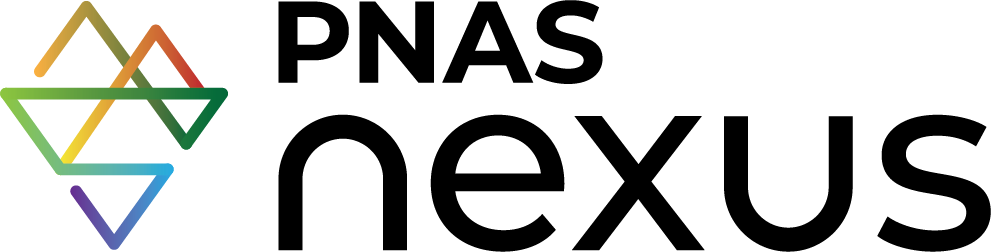


**Supplementary information for**

Ribosomal dysregulation: A conserved pathophysiological mechanism in human depression and mouse chronic stress.

Xiaolu Zhang^1*^, Mahmoud Ali Eladawi^2*^, William George Ryan^2^, Xiaoming Fan^3^, Stephen Prevoznik^2^, Trupti Devale^4^, Barkha Ramnani^4^, Krishnamurthy Malathi^4^, Etienne Sibille^5,6^, Robert Mccullumsmith^2,7^, Toshifumi Tomoda^5^, Rammohan Shukla^8 †^

**Table of Contents**

[Supplementary table-legends and notes 2](#_Toc133222829)

[Supplementary figures, legends, and notes: 3](#_Toc133222831)

[References 8](#_Toc133222832)

Supplementary table-legends and notes**:**

**Table S1: Differentially expressed genes (DEGs) identified in various studies investigating major depressive disorder (MDD) and experimental systems of chronic stress.** The DEGs from different studies are shown in separate Excel sheets. The human postmortem MDD data was obtained from Labonte *et al.*, Ramaker *et al.*, and Shukla *et al*. (1-3). The data for mice exposed to chronic variable stress (CVS) was obtained from Labonte *et al. (1)*, and the data for mice exposed to multimodal chronic stress (mCRS) was obtained from Weger *et al.* (4). Additionally, the table includes DEGs from mouse primary neuronal cultures exposed to dexamethasone in the absence or presence of RU-486 performed in the present study. The identified genes are considered differentially expressed based on their statistical significance (p-value or q-value < 0.05) compared to control conditions.

**Table S2:** **Enrichment of gene families in different discovery and validation datasets shown in Fig. 1.** The sheet named “HGNC_Human” and “HGNC_Mouse” details the gene list associated with different gene families in humans and mice, respectively. The list was curated from the HGNC database (5). The sheet named “Human_Enrichment” and “Mouse_Enrichment” shows the results of the hypergeometric analysis test used to assess the significant overlap between the “HGNC_Human” and “HGNC_Mouse” gene lists and discovery (Labonte *et al. (1)*) and validation (Ramaker *et al.* and Weger *et al. (2, 4))* datasets shown in Fig. 1. The -log10(p-value) is presented, and a value greater than 1.3 (i.e., -log10(0.05)) is considered significantly enriched.

**Table S3: Pathway Enrichment Analysis Details**

**Sheets 1 and 2** contain lists of genes correlated with RPG-seeds in human **(hs_SeedGene-Correlates)** and mouse **(hs_SeeGene-Correlates)**, respectively.

Sheet named “Fig. 2” provides details of the pathways shown in Fig. 2. Genes correlated with RPG-seeds were used to perform the pathway analysis. The -log10(q-value) is presented, and a value greater than 1.3 (i.e., -log10(0.05)) is considered significantly enriched. Green and yellow colors indicate pathways associated with negative and positive RPG-seed correlates, respectively.

Sheet named “Fig. 3” provides details of the pathway shown in Fig. 3. The -log10(q-value) is presented, and a value greater than 1.3 (i.e., -log10(0.05)) is considered significantly enriched.

Sheet named “Fig. S1” provides the results of the Gene Set Enrichment Analysis (GSEA (6)) performed with the Shukla *et al.* (3) dataset. The values in columns C and D represent the GSEA-based normalized enrichment score, which were used as coordinates to plot Fig. S1B. The truth table represents different pathway themes, such as presynapse, post synapse, adaptive and innate immunity, ribosomes, and glucocorticoid stimulus response, which are shown as colored dots and were enriched in either the episode or remission state.

**Table S4: Differentially expressed genes (DEGs) from a study on resilient and susceptible phenotypes against chronic social defeat stress (CSDS) and imipramine and ketamine responders and non-responders.** The DEGs from different studies are presented in individual Excel sheets. All data were sourced from Bagot et al. (7). Genes were considered differentially expressed if they showed statistical significance (p-value < 0.05) when compared to control conditions for resilient and susceptible phenotypes and when compared to CSDS-susceptible for responders and non-responders to imipramine and ketamine treatment.

Table S5: **Primer lists and metadata**

Sheet named “Primer Sequences” lists the primers used for qPCR quantification of ribosomal protein genes in MDD patients and CVS marker genes in mouse neuronal cell culture exposed to dexamethasone in the presence or absence of RU-486.

The sheets named "Labonte" and "Ramaker" provide the sample information from the two studies utilized in this study.

Supplementary figures, legends, and notes:

**Fig. S1:** **Reversibility of RPG dysregulation: A) Left:** The volcano plot shows RPG downregulation and RP-pseudogene upregulation during MDD episodes. **Right:** During remission from MDD, the pattern of RPG downregulation and RP pseudogene upregulation is reversed. As GSEA analysis (B) uses a rank-based profile to perform enrichment, it can reveal subtle changes associated with the phenotype that may not be significant at a p-value threshold of <0.05. Therefore, results with a significance threshold of >0.05 but <0.1 are also shown. **B)** Gene-set enrichment analysis of the episode (black dots) and remission state (blue dots) is shown in quadrant form. The four quadrants display distinct combinations of up- and downregulation during the two states. It is noteworthy that known synaptic and immune changes are downregulated and upregulated during episodes, respectively. Ribosome-related pathways were downregulated during the episode and upregulated during remission.

**Fig. S2: RPG and RP-Pseudogene Expression in CSDS-Exposed Animals and Antidepressant Responders.** (A-B) Distribution of RPG and RP-pseudogenes in resilient (A) and susceptible (B) animals subjected to CSDS. (C-D) Distribution of RPG and RP-pseudogenes in CSDS-susceptible mice exposed to imipramine and segregated into imipramine responders (C) and non-responders (D). Both groups of animals showed downregulation of RPGs, consistent with the core results using the CVS paradigm. (E-F) Distribution of RPG and RP-pseudogenes in CSDS-susceptible mice exposed to ketamine and segregated into Ketamine responders (E) and non-responders (F). Both responders and non-responders showed attenuated expression of both RPGs and RP-pseudogenes.

**Fig. S3: Validation of RPG dysregulation at the protein level:** Differential expression (p-value < 0.05) data obtained from the prefrontal cortex of resilient and susceptible rats exposed to eight weeks of chronic mild stress (CMS) (8) validates the dysregulation of RPGs observed in the core CVS-based results. Notably, the upregulated ribosomal proteins were more prominent than the downregulated ones in both resilient and susceptible animals. This difference may be attributed to several factors, including species differences and variations in allostatic loads between the CMS and CVS paradigms, with CVS involving more severe stressors. Additionally, it is possible that the downregulation of RPGs is driven by the upregulation of ribosomal proteins in a feedback manner. In this case, the excess ribosomal proteins (and thus upregulated) may be present in other organelles, such as lysosomes (ribophagy) and stress granules, for subsequent homeostasis processing.


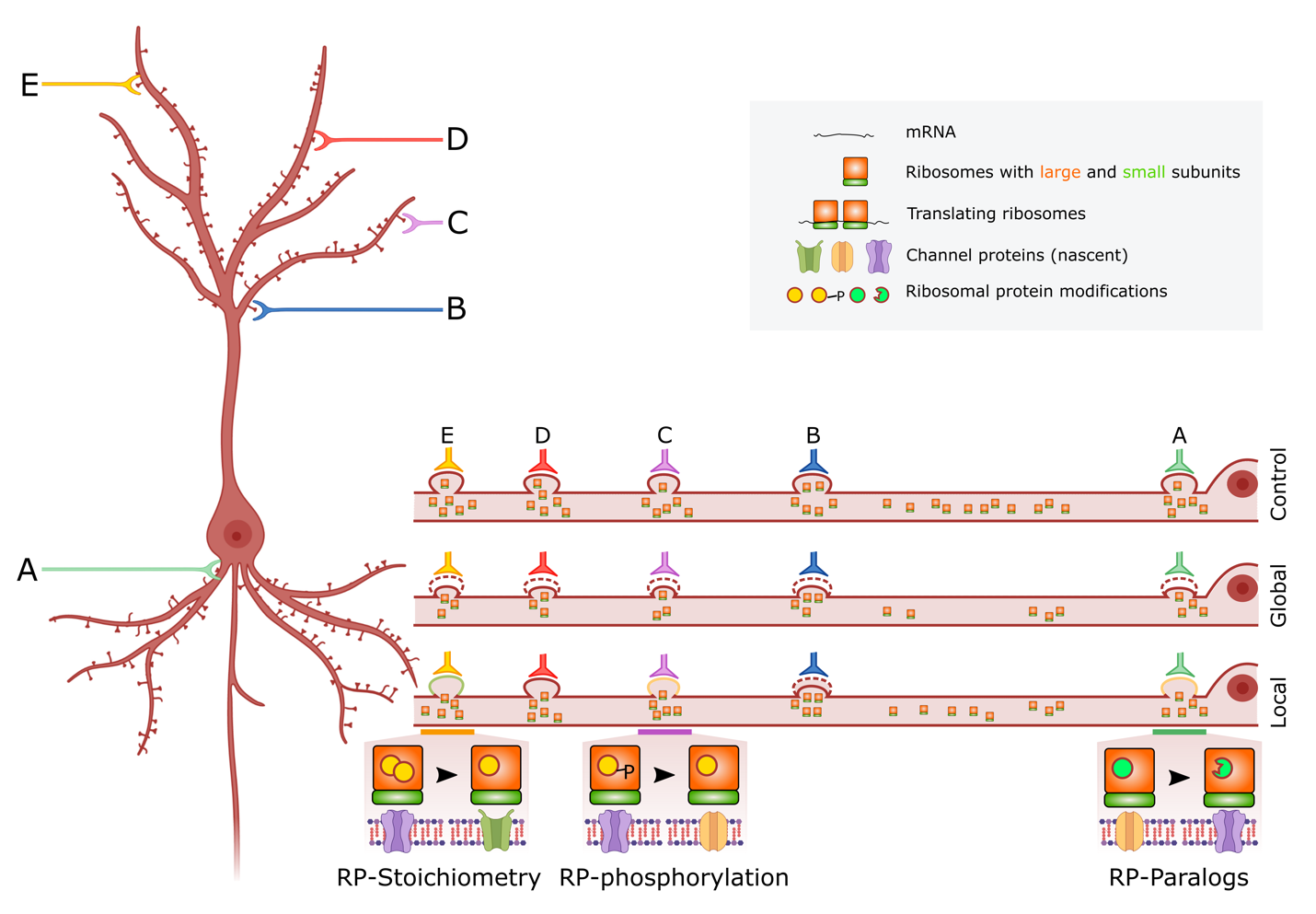


**Fig. S4: Potential mechanisms by which RPG downregulation could affect synaptic inputs (A through E).** **Global (location non-specific):** RPG downregulation may result in decreased ribosome production, which may reduce synthesis of synaptic proteins, resulting in decreased synaptic weight overall. **Local (location specific):** RPG downregulation may also change ribosome composition so that a few RPs are either removed, altered, or replaced by other RPs. These changes may result in the production of specialized ribosomes, which can alter synaptic protein translation in a cellular compartment-specific manner. Reduced ribosome production can accompany the production of specialized ribosomes, in which case reduced synaptic weight can also be observed locally.

**Fig. S5: Variance explained by each variable in a gene-expression experiment.** The figure shows a genome-wide violin plot depicting the distribution of variance explained by each variable across all genes in a previously deposited dataset by Labonte *et al (1).* and *Ramaker et al (2),* using variancePartion (9) package in R*.* The top four variables that explain the most variation in the gene-expression profiles were regressed out during the differential expression analysis. To account for variables such as batch and medication which were not provided in the deposited data, surrogate variable analysis (SVA) package in R (10) was used to summarize these variables along with two surrogate variables that account for unknown sources of variability. RIN: RNA integrity number, PMI: postmortem interval, COD: Cause of death.

References**:**

1. B. Labonté *et al.*, Sex-specific transcriptional signatures in human depression. *Nat Med* **23**, 1102-1111 (2017).

2. R. C. Ramaker *et al.*, Post-mortem molecular profiling of three psychiatric disorders. *Genome Medicine* **9**, 72 (2017).

3. R. Shukla *et al.*, Molecular characterization of depression trait and state. *Mol Psychiatry* 10.1038/s41380-021-01347-z (2021).

4. M. Weger *et al.*, Mitochondrial gene signature in the prefrontal cortex for differential susceptibility to chronic stress. *Scientific Reports* **10**, 18308 (2020).

5. R. L. Seal *et al.*, Genenames.org: the HGNC resources in 2023. *Nucleic Acids Res* 10.1093/nar/gkac888 (2022).

6. A. Subramanian *et al.*, Gene set enrichment analysis: A knowledge-based approach for interpreting genome-wide expression profiles. *Proceedings of the National Academy of Sciences* **102**, 15545 (2005).

7. R. C. Bagot *et al.*, Circuit-wide Transcriptional Profiling Reveals Brain Region-Specific Gene Networks Regulating Depression Susceptibility. *Neuron* **90**, 969-983 (2016).

8. W. Liao *et al.*, Chronic mild stress-induced protein dysregulations correlated with susceptibility and resiliency to depression or anxiety revealed by quantitative proteomics of the rat prefrontal cortex. *Translational Psychiatry* **11**, 143 (2021).

9. G. E. Hoffman, E. E. Schadt, variancePartition: interpreting drivers of variation in complex gene expression studies. *BMC Bioinformatics* **17**, 483 (2016).

10. J. T. Leek, W. E. Johnson, H. S. Parker, A. E. Jaffe, J. D. Storey, The sva package for removing batch effects and other unwanted variation in high-throughput experiments. *Bioinformatics* **28**, 882-883 (2012).
